# Screening of different species reveals cat hepatocytes support HBV infection

**DOI:** 10.1371/journal.ppat.1013390

**Published:** 2025-08-04

**Authors:** Zaichao Xu, Kaitao Zhao, Jingjing Wang, Lu Zhang, Jiatong Yin, Nijing Chen, Sijia Chen, Gaihong Zhao, Mengfei Wang, Tailai Xin, Chengliang Zhu, Xiaoming Cheng, Yuchen Xia

**Affiliations:** 1 State Key Laboratory of Virology and Biosafety, Hubei Provincial Research Center for Basic Biological Sciences and Hubei Province Key Laboratory of Allergy and Immunology, Institute of Medical Virology, TaiKang Center for Life and Medical Sciences, TaiKang Medical School, Wuhan University, Wuhan, China; 2 Department of Pathology, Center for Pathology and Molecular Diagnostics, Hubei Clinical Center and Key Laboratory of Intestinal and Colorectal Diseases, Zhongnan Hospital of Wuhan University, TaiKang Medical School, Wuhan University, Wuhan, China; 3 Department of Clinical Laboratory, Renmin Hospital of Wuhan University, Wuhan, China; 4 Pingyuan Laboratory, Henan, China; University of Pittsburgh School of Medicine, UNITED STATES OF AMERICA

## Abstract

Hepatitis B virus (HBV) remains a major public health challenge, with nearly 300 million chronic infections, yet research is hindered by the lack of suitable animal models. This study aimed to identify HBV-susceptible species and establish a novel infection model. Primary hepatocytes from humans, cats, rabbits, Syrian hamsters, Siberian hamsters, guinea pigs, bulls, goats, pigs, cynomolgus macaques, and dogs were assessed for HBV entry using hepatitis D virus (HDV) infection. HBV relaxed circular DNA (rcDNA) transfection was performed to evaluate its repair into covalently closed circular DNA (cccDNA). HBV infection assays were conducted *in vitro*. Results showed that primary hepatocytes from humans and cats were susceptible to HDV, suggesting their potential to support HBV entry. All tested hepatocytes converted rcDNA into cccDNA, confirming their ability to complete early HBV replication steps. Notably, cat hepatocytes uniquely supported HBV infection, displaying time-dependent viral replication marker expression. Cat hepatocytes also responded to antiviral treatments, underscoring their relevance for drug evaluation. This study provides the first evidence that cats can support HBV infection *in vitro*, offering a promising new platform for HBV research and antiviral development.

## Author summary

The lack of suitable animal models has significantly hindered HBV basic research and drug development. Here, we investigated the susceptibility of various species to HBV infection and revealed that cat hepatocytes support HBV infection and were responsive to antiviral treatments *in vitro*. This study provides the first evidence that cats can support HBV infection *in vitro*, offering a promising new platform for basic and translational HBV research.

## Introduction

Hepatitis B virus (HBV) infection poses a significant burden on global public health, with approximately 296 million chronically infected individuals worldwide [1]. There are two classes of treatments approved for chronic HBV infection, including interferon alpha (IFN-α) and nucleos(t)ide analogues (NAs) [2]. IFN-α is an immunomodulatory drug and is limited by its low response rate and severe side effects [3]. NAs can efficiently suppress viral replication but have little or no effect on HBV clearance, necessitating long term usage [4]. While both treatments are effective to some extent, they rarely lead to a cure for HBV infection.

HBV is a small, enveloped DNA virus that specifically targets human hepatocytes. It enters into hepatocytes via specific interactions with human sodium-taurocholate cotransporting polypeptide (hNTCP) [5]. Following internalization and uncoating, the viral relaxed circular DNA (rcDNA) is released into the nucleus, where it forms viral minichromosome-covalently closed circular DNA (cccDNA) [6]. This cccDNA serves as the template for the transcription of viral RNAs, which are subsequently transported to the cytoplasm and translated into viral proteins [7–10]. Among the viral transcripts, the 3.5 kb pre-genomic RNA (pgRNA) also serves as the template for rcDNA synthesis via reverse transcription catalyzed by HBV polymerase inside the capsid. The newly formed nucleocapsids are either recycled back to the nucleus to maintain the cccDNA pool or enveloped and released through multiple vesicle bodies as progeny virions to infect other cells [11].

The lack of suitable animal models has significantly hindered HBV basic research and drug development. HBV exhibits a highly restricted species tropism. Chimpanzees are the only non-human primate fully susceptible to HBV infection but they are no longer available for research [12]. Tree shrews are the only nonprimate animals can be experimentally infected with HBV [13]. However, they are limited by difficulties in breeding and the complex pre-handling prior to HBV infections [14]. Although rhesus macaques are naturally resistant to HBV infection, viral vector-mediated and liver-specific transgenic expression of hNTCP on hepatocytes renders them susceptible to HBV [15–17]. Mice are extensively used for studying human diseases due to their well-characterized genetic information and ease of manipulation. However, they cannot be infected with HBV due to the barrier in viral entry and cytoplasmic HBV nucleocapsids disassembly, as we recently reported [18]. Although varied animal models mimicking certain aspects of HBV life cycle have been established, an HBV infectable animal model is still needed.

Our study investigated the susceptibility of various species to HBV infection. Using isolated primary cells, we found cat hepatocytes support HBV infection and were responsive to antiviral treatments, highlighting the potential of cats as a candidate for developing a robust HBV infection animal model.

## Results

### The ability of various primary hepatocytes to facilitate viral entry

**Fig 1:**
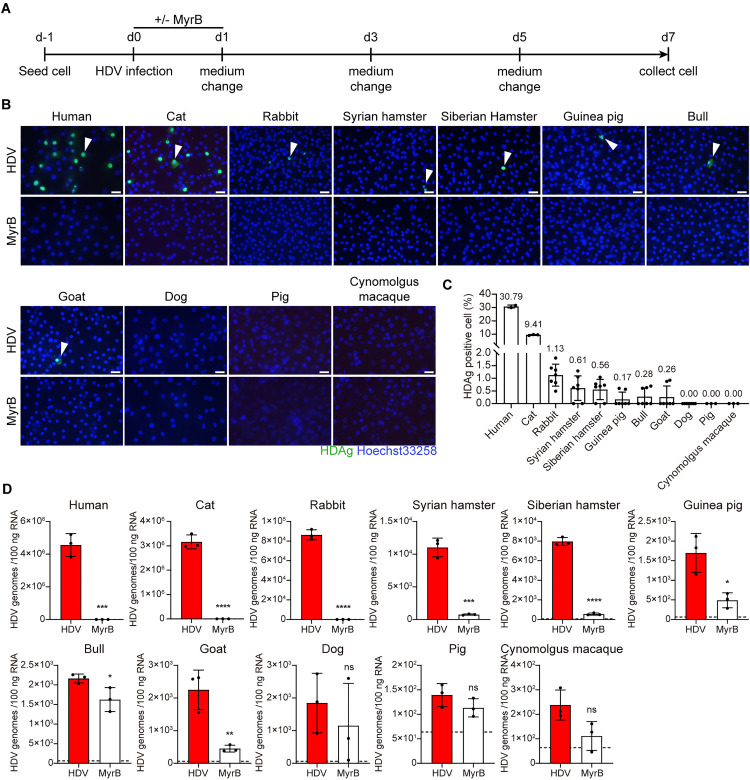
The ability of various primary hepatocytes to facilitate viral entry. (A) Schematic representation of the experimental setting. Multiple primary hepatocytes from different species were seeded in 12-well plates and infected with 200 HDV genomes per cell (MOI = 200) in the presence of 4% PEG8000 with or without 500 nM MyrB. One day post-infection, cells were washed three times with PBS and changed with fresh medium, the supernatants were collected at indicated time points. (B) Cells were fixed in 4% paraformaldehyde for the detection of HDAg by Immunofluorescence staining. HDAg positive cells were labeled with a white arrow. Scale bar = 50 µm. (C) Representative fields were randomly selected for imaging, and the proportion of positive cells was calculated. (D) Cells were harvested at 7 dpi and extracted RNA for the detection of HDV RNA. Dotted line represents assay limit of detection. Data was presented as mean ± SD. * p < 0.05, ** *p* < 0.01, *** *p* < 0.001, and **** *p* < 0.0001.MOI, multiplicity of infection. dpi, days post-infection.

Although intensive works have been made in establishing HBV animal models, it remains unclear whether hepatocytes from other species can support HBV infection. To address this, we first investigated the capability of primary hepatocytes from various species to mediate viral entry. HDV, a satellite virus of HBV that shares the same envelope composition [19], was used as a surrogate to test infectivity. Freshly isolated or cryopreserved primary hepatocytes from humans, cats, rabbits, Syrian hamsters, Siberian hamsters, guinea pigs, bulls, goats, pigs, cynomolgus macaques, and dogs were authenticated by their cytochrome c oxidase subunit I (COI) gene sequences (S1 and S2 Figs and S1 and S2 Tables). All primary hepatocytes were infected with HDV at a multiplicity of infection of 200 (MOI = 200). Myrcludex B (MyrB, also named Bulevirtide), a peptide specifically inhibits the interaction between HBV-derived preS1 and hNTCP [20], was used as a control (Fig 1A). Immunofluorescence revealed that the expression of HDAg was detectable in primary hepatocytes from humans, cats, rabbits, Syrian hamsters, Siberian hamsters, guinea pigs, bulls, and goats, which were effectively blocked by MyrB (Fig 1B). However, except human and cat, the rates of HDAg positive cells in other species were only between 0.17-1.13%, suggesting their limited susceptivity to HDV infection (Fig 1C). Along the same line, qPCR analyses demonstrated highly positive HDV RNA in humans and cats, and weak signals in rabbits, Syrian hamsters, Siberian hamsters, guinea pigs, bulls, and goats hepatocytes (Fig 1D).

**Fig 2:**
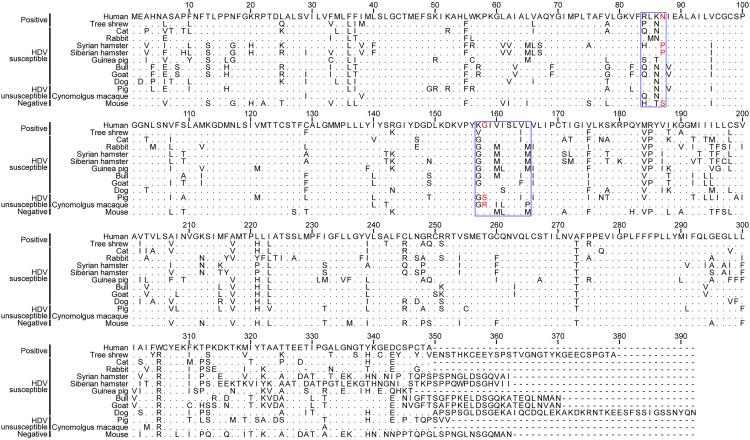
Alignment of NTCP sequences from different species. NTCP amino acid sequences from human (Protein ID. NP_003040.1), tree shrew (Protein ID. AFK93890.1), cat (Protein ID. XP_003987831.1), rabbit (Protein ID. NP_001076237.1), Syrian hamster (Protein ID: XP_005072871.1), Siberian hamster (Protein ID: XWU18636.1), guinea pig (Protein ID. XP_005008790.2), bull (Protein ID. NP_001039804.1), goat (Protein ID. XP_005686078.2), dog (Protein ID. XP_537494.2), pig (Protein Protein ID. XP_001927730.1), cynomolgus macaque (Protein ID. ALX38777.1), and mouse (Protein ID. AAH94023.1) are aligned using the alignment function of MEGA11 (Mega Limited). HBV-susceptible hosts (human and tree shrew) and HBV-unsusceptible hosts (mouse) are used as positive and negative reference, respectively. Residues 84-87 and 157-165 are boxed with blue. Residues 87 and 158 are labeled in red.

Since NTCP mediates the entry of both HBV and HDV, we analyzed its amino acid sequence across various species. Among them, the NTCP amino acid sequence of Siberian hamster was cloned and sequenced (S3 Table). Two key functional domains of NTCP, spanning amino acids (aa) 84–87 and 157–165, were highlighted (Fig 2). Notably, the G158 mutation in NTCP from cynomolgus macaques and pigs has been reported as a critical restriction site for HBV infection [21,22]. However, aside from cynomolgus macaques and pig NTCP, the key aa 158 in NTCP from other species is identical to that of humans (Fig 2).

Together, these findings indicate that primary hepatocytes from humans and cats can efficiently support HDV infection, suggesting their potential to support HBV entry.

### Cross-species analysis of rcDNA repair and cccDNA formation in primary hepatocytes

**Fig 3:**
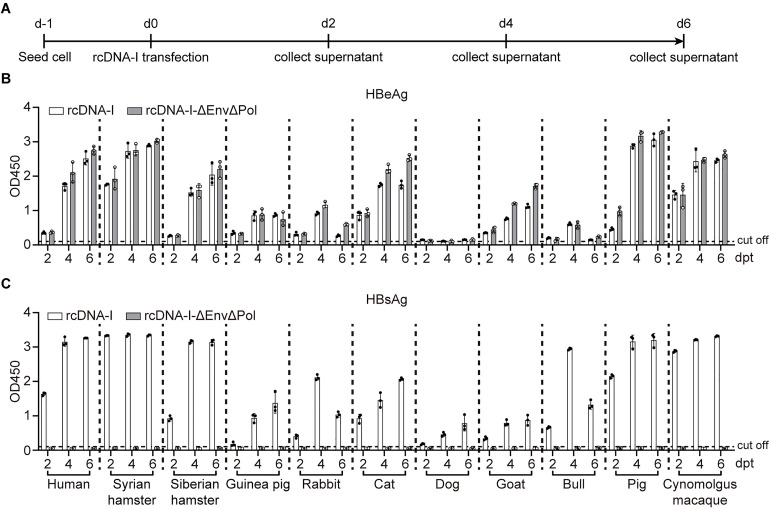
Cross-Species analysis of rcDNA repair and cccDNA formation in primary hepatocytes. (A) Schematic representation of the experimental setting. Multiple primary hepatocytes from different species were seeded in 12-well plates and transfected with rcDNA-I, the supernatants were collected at 2-, 4-, and 6 days post-transfection (dpt). (B-C) Secreted HBeAg and HBsAg in the supernatants at indicated time points were determined by ELISA.

As cccDNA formation is the key step of HBV infection, we next evaluated the repair process from rcDNA to cccDNA. To simulate this process, rcDNA repair intermediates (rcDNA-I) were prepared as described previously [23,24], and transfected into primary hepatocytes (Fig 3A). As a control, rcDNA-I-ΔEnvΔPol, containing deletion mutations in HBV envelope proteins and polymerase, was used to prevent pgRNA reverse transcription and viral egress. When wild-type rcDNA-I was transfected into primary hepatocytes from all the species, both HBeAg and HBsAg were detected in the cell culture supernatants in a time-dependent manner. In contrast, transfection with rcDNA-I-ΔEnvΔPol resulted in the secretion of only HBeAg, indicating that the intermediate rcDNA-I was successfully repaired to form cccDNA in these cells (Fig 3B and 3C).

These findings demonstrate that primary hepatocytes from multiple species are capable of supporting the repair process from rcDNA to cccDNA.

### Susceptibility of primary hepatocytes from various species to HBV infection

**Fig 4:**
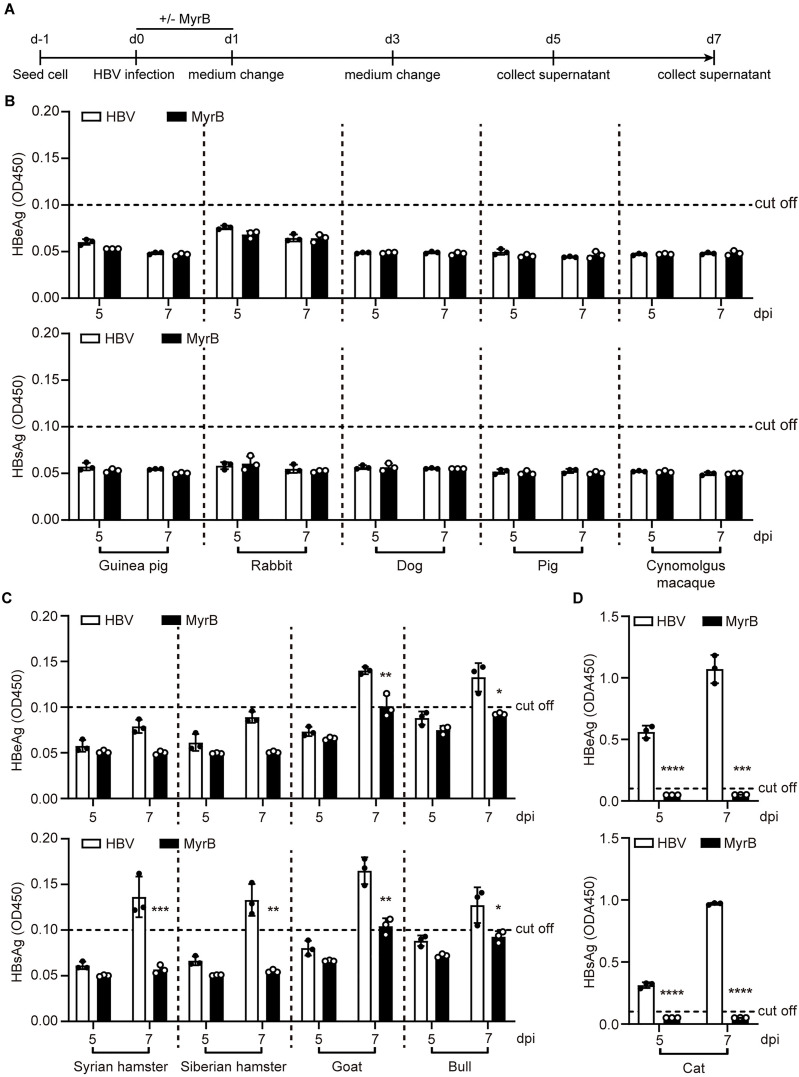
Susceptibility of primary hepatocytes from various species to HBV infection. (A) Schematic representation of the experimental setting. Multiple primary hepatocytes from different species were seeded in 12-well plates and infected with HBV at 800 genomes per cell (MOI = 800) in the presence of 4% PEG8000 with or without 500 nM MyrB. One day post-infection, cells were washed three times with PBS and changed with fresh medium, the supernatants were collected at 5 dpi and 7 dpi. (B-D) Secreted HBeAg and HBsAg in the supernatants at 5 dpi and 7 dpi were determined by ELISA. Data was presented as mean ± SD. * p < 0.05, ** *p* < 0.01, *** *p* < 0.001, and **** *p* < 0.0001. MOI, multiplicity of infection. dpi, days post-infection.

We next investigated the susceptibility of primary hepatocytes from various species to HBV infection. Primary hepatocytes were directly infected with HBV (Fig 4A). The results showed that hepatocytes from guinea pigs, rabbits, dogs, pigs, and cynomolgus macaques were insensitive to HBV infection, as no HBV antigens were detected (Fig 4B). In primary Syrian hamsters, Siberian hamsters, goats, and bulls hepatocytes, HBsAg and HBeAg levels remained low and close to the detection threshold at 7 days post-infection (dpi). However, these weak signals were suppressed by MyrB treatment, indicating a very limited infection efficiency (Fig 4C). Interestingly, primary cat hepatocytes exhibited positive and time-dependent secretion of HBV antigens from 5 dpi to 7 dpi, which was effectively blocked by MyrB (Fig 4D). These findings demonstrate that primary cat hepatocytes are capable of supporting HBV infection.

### Comprehensive analysis of the HBV life cycle in primary cat hepatocytes

**Fig 5:**
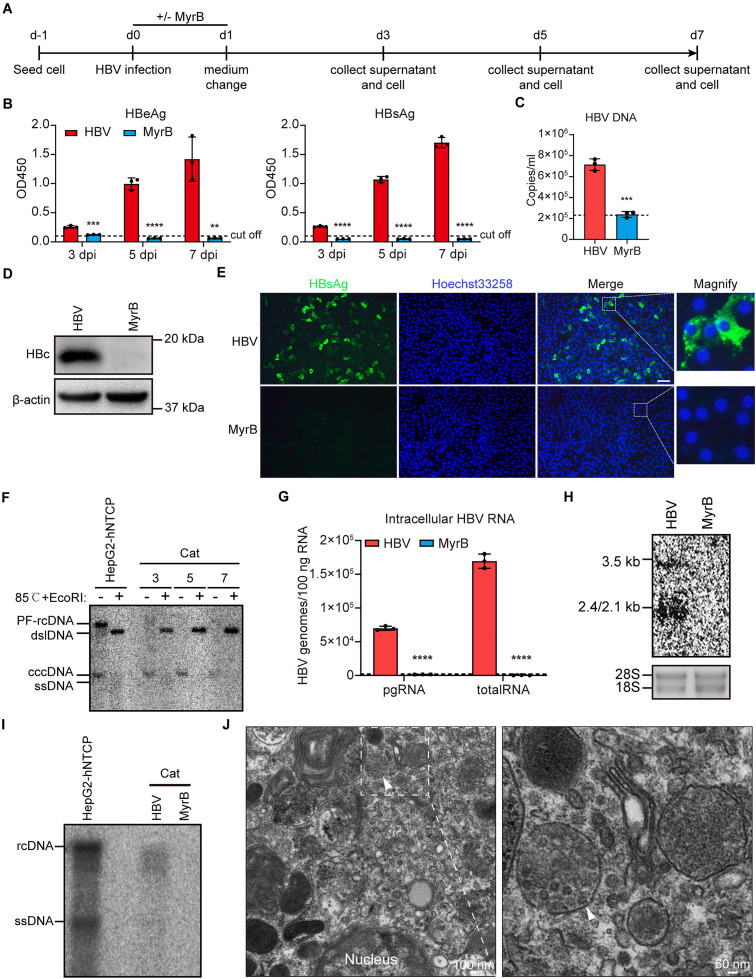
Comprehensive analysis of the HBV life cycle in primary cat hepatocytes. (A) Schematic representation of the experimental setting. Primary cat hepatocytes were seeded in 6-well plates and infected with 2000 HBV genomes per cell (MOI = 2000) in the presence of 4% PEG8000 with or without 500 nM MyrB. (B) Secreted HBeAg and HBsAg in the supernatants at 3 dpi, 5 dpi, and 7 dpi were determined by ELISA. (C) HBV DNA of the cell culture supernatants at 7 dpi was detected by qPCR using an HBV viral DNA quantitative fluorescence diagnostic kit. (D) The expression of HBc protein was detected by western blotting. β-actin was used as an internal control. (E) The expression and localization of HBsAg protein was detected by immunofluorescence staining. Scale bar = 100 µm. (F) Cellular DNA was Hirt extracted and denatured at 85°C, treated with EcoRI restriction nuclease, and subjected to Southern blotting using ^32^P labeled HBV probes. The Hirt DNA samples from HBV-infected HepG2-hNTCP cells served as the positive control. (G) Cellular total RNA was extracted and reverse-transcribed, and HBV pgRNA and total RNA were quantified by RT-qPCR using HBV-specific primers. (H) Cellular total RNA was extracted and then subjected to Northern blotting assay using ^32^P labeled HBV probes. Ribosomal RNA 18S and 28S served as the loading control. (I) Cellular HBV core-associated DNA was extracted and then subjected to Southern blotting assay using ^32^P labeled HBV probes. The DNA samples from HBV-infected HepG2-hNTCP cells served as the positive control. (J) Transmission electron microscopy (TEM) was used for cellular ultrastructural examination of MVBs containing HBV particles (white arrow). Dotted line represents assay limit of detection. Data was presented as mean±SD. * p < 0.05, ** *p* < 0.01, *** *p* < 0.001, and **** *p* < 0.0001. MOI, multiplicity of infection. HBc, HBV core protein; pgRNA, pre-genomic RNA; rcDNA, relaxed circular DNA; dslDNA, double-strand linear DNA; ssDNA, single-strand DNA; PF-rcDNA, protein-free rcDNA; cccDNA, covalently closed circular DNA; dpi, days post-infection.

To further confirm the susceptibility of cat hepatocytes to HBV, we evaluated different HBV replication markers after infection (Fig 5A). As the results shown, increased levels of secreted HBeAg and HBsAg were observed in the culture supernatants of the HBV-infected cat hepatocytes, which could be completely blocked by MyrB (Fig 5B). HBV particle secretion was accessed by HBV DNA in supernatant (Fig 5C). Western blot analysis confirmed intracellular HBc expression (Fig 5D), while immunofluorescence revealed the expression and localization of HBsAg (Fig 5E).

To determine whether cccDNA was established in primary cat hepatocytes, intracellular Hirt DNA was extracted and analyzed via Southern blotting. The presence of cccDNA was confirmed by heating the DNA sample to 85°C, which denatures rcDNA and dslDNA into single-stranded DNA, but not cccDNA. Subsequent treatment with EcoRI converted cccDNA into dslDNA. As shown in Fig 5F, Hirt DNA from HBV-infected primary cat hepatocytes showed a distinct cccDNA band that was converted into dslDNA after these treatments, with the cccDNA content increasing until day 5 and then remaining stable. Intracellular HBV transcripts, including pgRNA and total RNA, were quantified using RT-qPCR (Fig 5G). Northern blotting identified pgRNA (3.5 kb) and mRNAs encoding surface proteins (2.1 kb and 2.4 kb) (Fig 5H). Southern blotting confirmed HBV core-associated DNA (Fig 5I). To further investigate the assembly of mature HBV particles in primary cat hepatocytes, the ultrastructure of multivesicular bodies (MVBs) was examined using transmission electron microscopy. MVBs containing HBV particles were observed in HBV-infected primary cat hepatocytes (Fig 5J).

These findings demonstrate that primary cat hepatocytes support the complete HBV life cycle, including natural infection, cccDNA formation, transcription, replication, packaging, and secretion.

### Comparison of human and cat hepatocytes in supporting HBV infection

**Fig 6:**
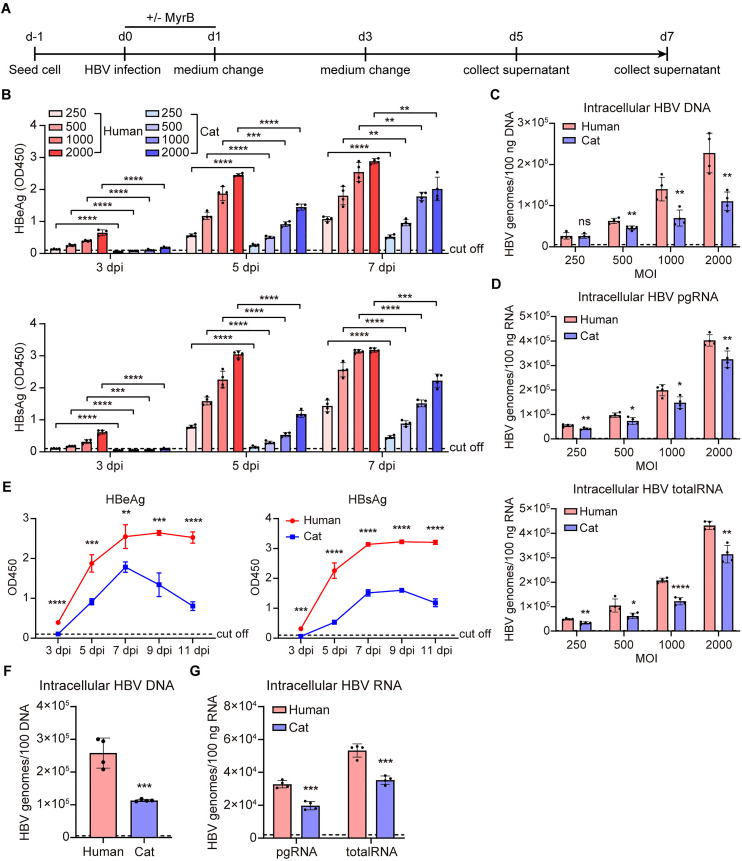
Primary human hepatocytes support higher HBV infection and replication. (A) Schematic representation of the experimental setting. Primary hepatocytes were seeded in 48-well plates and infected with HBV (MOI = 250, 500, 1000, and 2000) in the presence of 4% PEG8000 with or without 500 nM MyrB. (B) Secreted HBeAg and HBsAg in the supernatants at 3 dpi, 5 dpi, and 7 dpi were determined by ELISA. (C) Intracellular DNA was extracted at 7 dpi and detected by qPCR using an HBV viral DNA quantitative fluorescence diagnostic kit. (D) Cellular total RNA was extracted at 7 dpi and reverse-transcribed, HBV pgRNA and total RNA were quantified by RT-qPCR using HBV-specific primers. (E-G) Secreted HBeAg and HBsAg (MOI = 1000) in the supernatants at indicated time points were determined by ELISA. Intracellular HBV DNA and RNA were detected by qPCR at 11 dpi. Dotted line represents assay limit of detection. Data was presented as mean ± SD. * p < 0.05, ** *p* < 0.01, *** *p* < 0.001, and **** *p* < 0.0001. MOI, multiplicity of infection. pgRNA, pre-genomic RNA; dpi, days post-infection.

Next, we compared the efficiency of HBV infection and replication between human and cat hepatocytes (Fig 6A). As shown, both HBeAg and HBsAg levels increased in the culture supernatants of the HBV-infected human and cat hepatocytes in a time- and dose-dependent manner (Fig 6B). Higher levels of intracellular HBV DNA and RNA were observed in human hepatocytes (Fig 6C and 6D). Accordingly, long-term monitoring showed lower HBV replication in cat hepatocytes (Fig 6E-6G). These results indicated that compared with primary human hepatocytes, primary cat hepatocytes support HBV infection and replication with lower efficiency.

**Fig 7:**
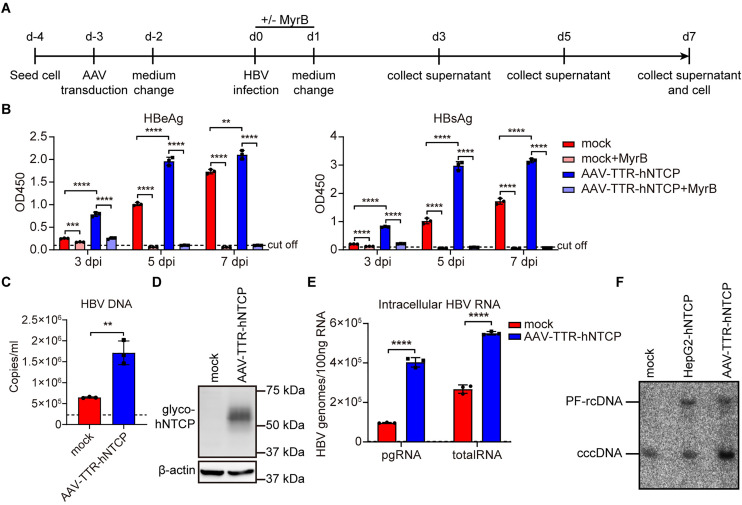
Enhanced HBV susceptibility in primary cat hepatocytes overexpressing hNTCP. (A) Schematic representation of the experimental setting. Primary cat hepatocytes were seeded in 6-well plates and infected with HBV at a multiplicity of infection of 2000 (MOI = 2000), or transduced with AAV-TTR-hNTCP (60000 vg/cell) in the presence of 4% PEG8000. 24 hours post-HBV infection or AAV transduction, cells were washed thrice with PBS and changed with fresh PMM. 72 hours post-AAV transduction, cells were infected with HBV (MOI = 2000). (B) Secreted HBeAg and HBsAg in the supernatants at indicated time points were determined by ELISA. (C) HBV DNA of the cell culture supernatants at 7 dpi was detected by qPCR using an HBV viral DNA quantitative fluorescence diagnostic kit. (D) The expression of hNTCP protein in primary cat hepatocytes overexpressing hNTCP or not was detected by western blotting at 7 dpi. β-actin was used as an internal control. (E) Cellular total RNA was extracted and reverse-transcribed, HBV pgRNA and total RNA were quantified by RT-qPCR using HBV-specific primers. (F) The Hirt DNA samples from 7 dpi were extracted and subjected to Southern blotting using ^32^P labeled HBV probes. Dotted line represents assay limit of detection. Data was presented as mean ± SD. * **p* *< 0.05, ** *p* < 0.01, *** *p* < 0.001, and **** *p* < 0.0001. MOI, multiplicity of infection. pgRNA, pre-genomic RNA; PF-rcDNA, protein-free rcDNA; cccDNA, covalently closed circular DNA; dpi, days post-infection; vg: viral genomes.

We next ectopically expressed hNTCP in primary cat hepatocytes and subsequently infected them with HBV (Fig 7A). Notably, hNTCP overexpression led to increased levels of HBV antigens and DNA in the cell culture supernatants (Fig 7B and 7C). Western blot analysis confirmed intracellular hNTCP expression in AAV-transduced cells (Fig 7D). Elevated levels of intracellular HBV RNA and cccDNA were further confirmed by RT-qPCR (Fig 7E) and Southern blotting (Fig 7F), respectively. These results indicate that hNTCP enhances HBV infection in primary cat hepatocytes.

### Evaluation of primary cat hepatocytes as a platform for testing anti-HBV drugs

**Fig 8:**
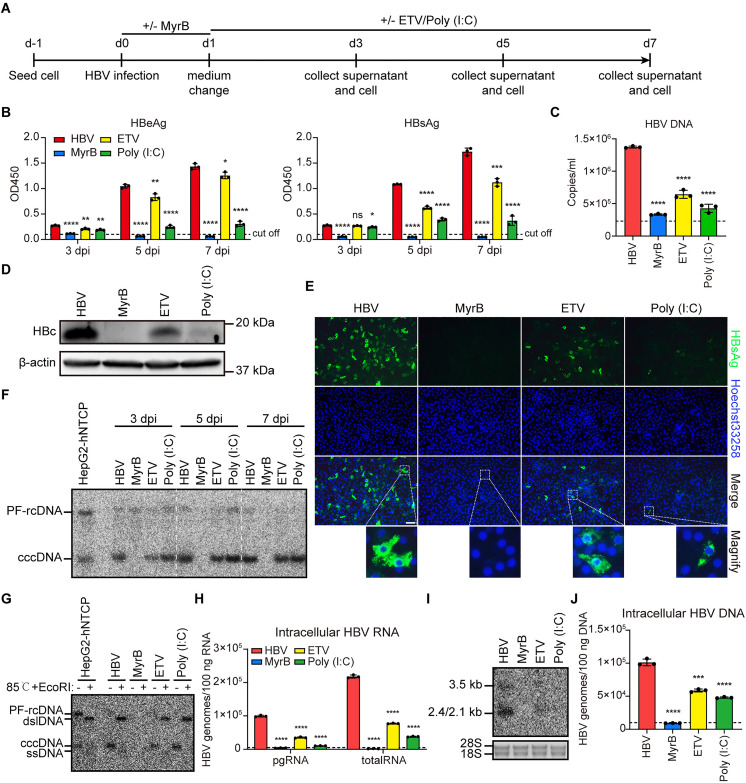
Evaluation of primary cat hepatocytes as a platform for testing anti-HBV drugs. (A) Schematic representation of the experimental setting. Primary cat hepatocytes were seeded in 6-well plates and infected with 2000 HBV genomes per cell (MOI = 2000) in the presence of 4% PEG8000 with or without 500 nM MyrB. One day post-infection, cells were treated with or without ETV (500 nM) and Poly (I:C) (1 μg/ml), and the supernatants and cells were collected at 3 dpi, 5 dpi, and 7 dpi. (B) Secreted HBeAg and HBsAg in the supernatants at indicated time points were determined by ELISA. (C) HBV DNA of the cell cultural supernatants at 7 dpi was detected by qPCR using an HBV viral DNA quantitative fluorescence diagnostic kit. (D) The expression of HBc protein in primary cat hepatocytes was detected by western blotting. β-actin was used as an internal control. (E) The expression of HBsAg protein in primary cat hepatocytes was detected by immunofluorescence staining. Scale bar = 100 µm. (F) Cellular DNA from different time points was Hirt extracted and then subjected to Southern blotting assay using ^32^P labeled HBV probes. The Hirt DNA samples from HBV-infected HepG2-hNTCP cells served as the positive control. (G) The Hirt DNA samples from seven days post-infection was denatured at 85°C, treated with EcoRI restriction nuclease, and subjected to Southern blotting using ^32^P labeled HBV probes. The Hirt DNA samples from HBV-infected HepG2-hNTCP cells served as the positive control. (H) Cellular total RNA was extracted and reverse-transcribed, and HBV pgRNA and total RNA were quantified by RT-qPCR using HBV-specific primers. (I) Cellular total RNA was extracted and then subjected to Northern blotting assay using ^32^P labeled HBV probes. Ribosomal RNA 18S and 28S served as the loading control. (J) Intracellular DNA was extracted and detected by qPCR using an HBV viral DNA quantitative fluorescence diagnostic kit. Dotted line represents assay limit of detection. Data was presented as mean ± SD. * p < 0.05, ** *p* < 0.01, *** *p* < 0.001, and **** *p* < 0.0001. MOI, multiplicity of infection. HBc, HBV core protein; pgRNA, pre-genomic RNA; rcDNA, relaxed circular DNA; dslDNA, double-strand linear DNA; ssDNA, single-strand DNA; PF-rcDNA, protein-free rcDNA; cccDNA, covalently closed circular DNA; dpi, days post-infection.

We next assessed whether primary cat hepatocytes are suitable for studying antiviral agents against HBV. HBV-infected cells were treated with MyrB, entecavir (ETV), and Poly (I:C), and various HBV replication markers in the supernatants and cells were analyzed (Fig 8A). Compared to the HBV-infected group, MyrB treatment completely blocked HBV infection, as indicated by undetectable HBV replication markers, including the secretion of HBeAg, HBsAg, and HBV particles in culture supernatants (Fig 8B and 8C). MyrB also suppressed intracellular HBc and HBsAg protein expression, as well as HBV cccDNA, transcripts, and DNA (Fig 8D-8J).

ETV, a reverse transcription inhibitor that targets HBV polymerase activity [25], significantly reduced HBV replication. Although a time-dependent increase in secreted HBeAg and HBsAg was observed during ETV treatment, their levels were markedly lower compared to untreated group (Fig 8B). HBV DNA levels in the supernatant were also significantly inhibited (Fig 8C). Western blotting and immunofluorescence revealed reduced intracellular HBc and HBsAg protein expression (Fig 8D and 8E). A time-dependent increase in cccDNA was detected, but it remained lower than control group (Fig 8F and 8G), suggesting a cccDNA amplification, probably through virus spreading or capsid recycling, similar to what we previously observed in stem cell derived hepatocytes-like cells [26]. RT-qPCR and Northern blotting further confirmed a reduction in HBV RNA levels (Fig 8H and 8I), while intracellular HBV DNA was significantly decreased (Fig 8J).

Poly (I:C), a synthetic double-stranded RNA mimetic and TLR3 ligand [27], also showed significant antiviral effects. Poly (I:C) treatment substantially reduced secreted HBV antigens, HBV DNA levels, and intracellular HBc and HBsAg protein expression (Fig 8B-8E). Although cccDNA reduction was marginal (Fig 8F and 8G), HBV transcripts and intracellular DNA were significantly inhibited (Fig 8H-8J).

These findings demonstrate that primary cat hepatocytes provide a robust platform for evaluating anti-HBV drugs.

## Discussion

Establishing an animal model for HBV infection presents significant challenges due to the species specificity of the virus and the complexity of the host-virus interactions. HBV naturally infects only humans and chimpanzees [12]. Most common laboratory animals like mice, rats, and rabbits are not susceptible to HBV due to the inability to support the viral life cycle [22,28]. While chimpanzees can be infected with HBV, ethical concerns, high costs, and their endangered status make them impractical for routine research [12]. Humanized liver mice are potential solutions, but these mice are expensive, technically challenging to produce, and have issues with engraftment stability [29]. A novel orthohepadnavirus species, known as capuchin monkey hepatitis B virus, has been identified in capuchin monkeys [30], and a chimeric simian-tropic HBV infection model has been established in marmoset cells [31]. Rhesus macaques cannot support HBV infection, whereas viral vector-mediated expression of hNTCP on hepatocytes *in vitro* and *in vivo* renders rhesus macaques permissive to HBV infection [15,16]. Further research suggested that liver-specific transgenic expression of hNTCP in rhesus macaques confers HBV susceptibility on primary hepatocytes [17]. Other animal models, such as the woodchuck and Pekin duck, are used for studying related hepadnaviruses, including woodchuck hepatitis virus and duck hepatitis B virus [32,33]. Moreover, a recent study demonstrated that species-specific NTCP orthologues from animals including woodchuck, ferret, aardvark, horse, rabbit, whale, big brown bat, cat, and rhinoceros can render HepG2 cells susceptible to HBV infection when expressed ectopically. Notably, primary horse hepatocytes were shown to be fully permissive to HBV infection [34]. Additionally, humanized hamster NTCP expressed in HepG2 cells has also been found to support HBV infection [35]. However, while these can provide insights, they do not fully replicate human HBV infection. Due to these challenges, developing an ideal HBV infection animal model is difficult. Cat supports HBV infection *in vitro*. This model may offer a new platform for the basic and clinical research of HBV.

NTCP is a bile acid transporter predominantly expressed on the basolateral membrane of hepatocytes. Beyond its physiological role, NTCP is uniquely exploited by HBV during viral infection [5]. The interaction and entry of HBV are facilitated by NTCP’s recognition of the myristoylated preS1 domain of the HBV large envelope protein, which serves as a critical determinant for viral attachment [36]. Previous studies have identified crucial regions in the NTCP sequence, including aa 84–87 and 157–165, as key determinants of HBV susceptibility across species. For example, in murine NTCP (mNTCP), residues 84–87 restrict productive HBV entry, whereas substituting these residues with their hNTCP counterparts (triple mutation 84/86/87) restores HBV infectivity in Huh7 cells [28,37]. The TM2-TM3 loop encompassing this region has been shown to bind the C-terminal portion of the preS1 domain [38]. Likewise, the expression of nonfunctional monkey or pig NTCP with humanized aa 157–167 renders them susceptible to HBV in HepG2 cells [5,39]. In addition, aa 158 of NTCP plays a pivotal role in facilitating HBV attachment to host cells, highlighting a mechanism by which HBV infection exerts positive selection pressure on this specific NTCP residue [40,41]. Notably, our findings align with data from tree shrew NTCP, which also supports HBV infection despite differences in this region. Combined with our HDV infection results, these observations underscore the critical roles of residue aa 87 and aa 158 in determining HBV susceptibility within these two regions.

Recently, domestic cat hepadnavirus (DCH), a novel hepadnavirus in cats, was identified in many countries and was associated with feline chronic hepatitis [42–45]. This DCH has a partially double-stranded, circular DNA genome of ∼3,200 bases in length. As observed with other Orthohepadnavirus species, the genome of DCH contains polymerase, surface, core, and X ORFs [42]. Notably, the preS1 sequence of DCH is genetically closer to human HBV than to woodchuck hepatitis virus or arctic ground squirrel hepatitis B virus, suggesting that DCH preS1 may function similarly to HBV preS1 [46]. Furthermore, HBV- and DCH-derived preS1 peptides efficiently bound to both human and cat NTCPs, with residue 158 of the NTCP proteins determining the species-specific binding of the DCH preS1 peptide. Along the same line, our study demonstrates that cat NTCP supports HBV entry.

The potential for inter-species transmission between human HBV and DCH is a subject of interest due to the genetic and functional similarities between the viruses, particularly in the preS1 sequence, which facilitates binding to NTCP receptors in both humans and cats [46]. Although this suggests a theoretical risk of cross-species infection, significant barriers such as species-specific immune responses, host cell factors, and the need for viral adaptation make such transmission uncommon. Nevertheless, the possibility raises concerns about zoonotic and reverse zoonotic transmission, particularly in scenarios involving close human-cat interactions or immunocompromised individuals [47,48]. This underscores the need for ongoing research and surveillance to better understand the host range of DCH, its potential to infect humans, and any implications for public health and veterinary medicine.

One limitation of the study is the *in vivo* experiment is not explored. Chronic HBV infection is associated with persistent viral replication, immune system evasion, and the development of liver diseases such as fibrosis, cirrhosis, and hepatocellular carcinoma [49]. Without studying these long-term effects, it’s unclear whether this model can accurately reflect the full spectrum of HBV-related diseases as seen in human patients. Furthermore, the immune response to HBV in humans involves a complex interplay between innate and adaptive immunity, including the activation of specific T cells, production of antibodies, and the role of cytokines and other immune modulators [50]. These responses can vary greatly across different species, influencing how the infection progresses and whether it becomes chronic. Despite these advancements, the study highlights the need for further *in vivo* validation and a more comprehensive understanding of the biological mechanisms involved in HBV cat model.

Our study provides the first evidence that cat supports HBV infection *in vitro*. This model may offer a new platform for the basic and clinical research of HBV. Using cats as an HBV infection model offers several advantages. Cats are more accessible and cost-effective than primates and have a longer lifespan than rodents, making them suitable for studying chronic HBV infection and liver pathology [51]. Their well-characterized immune system and susceptibility to other viruses also provide a valuable platform for exploring co-infections and immune responses [52,53]. Additionally, cats’ responsiveness to existing antiviral drugs makes them a practical model for testing new therapies [52]. These factors highlight their potential for advancing HBV research and treatment development.

## Materials and methods

### Ethics statement

Animals used in this study were treated in accordance with the guidelines on humane care, and the protocols were approved by the Ethic Committee of Animal Facility, Wuhan University.

### Cell culture

HEK293T and Huh7 cell lines were maintained in Dulbecco’s modified Eagle medium (DMEM) supplemented with 10% fetal bovine serum (Lonsera, Montevideo, Uruguay) and 1% penicillin/streptomycin (Gibco, Massachusetts, USA), and cultured at 37°C with 5% CO_2_. Primary human, cynomolgus macaque, pig, cat, goat, bull, and dog hepatocytes were obtained from Liver Biotechnology Co., Ltd (Shenzhen, China) and cultured according to the manufacturer’s protocol.

### Drugs

Bulevirtide (Myrcludex B) (Cat. No. HY-P3465), ETV (Cat. No. HY-13623), and dexamethasone phosphate disodium (Cat. No. HY-B1829A) were purchased from MedChemExpress (New Jersey, USA). Poly(I:C) (tlr-picw) was purchased from InvivoGen (California, USA).

### Primary hepatocytes isolation

Primary rabbit, Syrian hamster, Siberian hamster, and guinea pig hepatocytes were isolated from animals ethically sourced through approved institutional protocols. The isolation procedures, including the two-step perfusion method, were conducted *ex vivo* following euthanasia and in compliance with relevant animal welfare regulations as previously described before [18]. In brief, D-Hank’s balanced salt solution (Pricella, Wuhan, China) supplemented with 2 mM glutamine, 0.5% glucose, 25 mM 4-(2-hydroxyethyl)-1-piperazine ethanesulfonic acid (HEPES), and 2 mM ethylene glycol tetraacetic acid (EGTA) was firstly used to perfuse the liver. Then, the collagenase type IV (Merck, Darmstadt, German) containing buffer (D-Hank’s balanced salt solution supplemented with 2 mM glutamine, 0.5% glucose, 25 mM HEPES, 3 mM CaCl_2_, and 0.3 mg/ml collagenase type IV) was used to perfuse and digest the liver. PHs were washed with DMEM supplemented with 5% fetal bovine serum (Lonsera, Montevideo, Uruguay) and 1% penicillin/streptomycin (Gibco, Massachusetts, USA), then purified with percoll (Biosharp, Hefei, China) density gradient solutions. Purified hepatocytes were washed, collected, and then resuspended in primary hepatocytes plating medium (PPM) (Williams E medium supplemented with 10% fetal bovine serum, 1% penicillin/streptomycin, 1% ITS (Pricella, Wuhan, China), 10 ng/ml EGF, 2 mM L-glutamine, 18 μg/ml hydrocortisone, and 40 ng/ml dexamethasone). Hepatocytes were plated 1.5 × 10^5^ cells/cm^2^ on a culture well plate pretreated with 50 μg/ml rat tail collagen (Corning, New York, USA) overnight. After 6 hours, the cultures were changed with primary hepatocytes maintenance medium (PMM) (Williams E medium supplemented with 1% penicillin/streptomycin, 1% ITS, 10 ng/ml EGF, 2 mM L-glutamine, 18 μg/ml hydrocortisone, 40 ng/ml dexamethasone, and 1.8% DMSO).

### RcDNA repair intermediates production and transfection

Different rcDNA repair intermediates were prepared as described previously [23]. The minus and plus single strand DNA (+ssDNA and -ssDNA) used to synthesize rcDNA were generated from the plasmids pX330-(+)DNA and pX330-(-)DNA, respectively. The plasmids were digested with NheI and Nt.BspQI, and the ssDNA was separated by agarose gel electrophoreses and purified. The intermediate rcDNA-II was synthesized by mixing and annealing +ssDNA and -ssDNA at a molar ratio of 1:1. To generate rcDNA-I, the oligo 5′-biotin-GAAAAAGTTGCATGGTGCTGGTG and oligo 5′-rGrCrArArCrUrUrUrUrUrCrArCrCrUrCrUrGrCACGTCGCATGGAGACCACCGT (BGI Tech Solutions, Shenzhen, China) were ligated to rcDNA-II using T4 ligase (Thermo Fisher, Massachusetts, USA). The ligase was removed by phenol-chloroform extraction and the ligation product was purified. The ligation efficiency was determined as described previously [23]. rcDNA-I and rcDNA-II were digested by BsrDI and purified, then the products were mixed with an equal volume of 2x miRNA Deionized Formamide Gel Loading Buffer (Sangon Biotech, Shanghai, China) and heated and resolved on a 10% urea-PAGE gel electrophoresis. The Gel was finally stained with Gelred.

For rcDNA repair intermediates transfection, Lipofectamine 3000 (Thermo Fisher, Massachusetts, USA) was used according to the manufacturer’s instructions with some modifications. Lipofectamine 3000, P3000 regent, and rcDNA repair intermediates were mixed in a ratio of 3:2:1 (μl:μl:μg) and incubated at room temperature for 15 min. The mixture was then added to the cells. After 6 hours of transfection, the medium was removed and replaced with fresh culture medium and cultured further as indicated for the following experiment.

### HBV production and infection

HBV stocks were produced by the HepAD38 cell line with stable HBV replication [54]. Briefly, HepAD38 cell culture supernatants were harvested and filtered with 0.22 μm filter, then concentrated using 100-kDa Ultra Centrifugal Filters (Millipore, Massachusetts, USA) and subsequently purified by sucrose gradient ultracentrifugation using Ultracentrifuge L-100XP (Beckman Coulter Life Sciences, California, USA). HBV titers were titrated using Hepatitis B Viral DNA quantitative fluorescence diagnostic kit (Sansure Biotech, Changsha, China). Aliquoted HBV stocks were stored at -80°C.

For HBV infection, PH cells were infected with 800 or 2000 HBV genomes per cell (multiplicity of infection, MOI = 800 or 2000) in the presence of 4% PEG8000 (Sigma-Aldrich, Missouri, USA). One day post-infection, cells were washed three times with PBS and treated further as indicated for the following experiment.

### HDV production and infection

Hepatitis D virus (HDV) production was performed as described previously [55]. Briefly, the pSVLD3 plasmid containing the triploid HDV genome and the pT7HB2.7 plasmid containing the HBV surface protein gene were co-transfected into Huh7 cells. The cell culture supernatants were harvested and filtered with 0.22 μm filter, then concentrated using 100-kDa Ultra Centrifugal Filters (Millipore, Massachusetts, USA) and subsequently purified by sucrose gradient ultracentrifugation. The virus genome was extracted using RNApure Tissue & Cell Kit (Cowin Biotech, Beijing, China) and transcribed into cDNA by ReverTra Ace qPCR RT Master Mix (TOYOBO BIOTECH CO., Osaka, Japan). HDV RNA was detected by RT-qPCR using LightCycler 480 (Roche Diagnostics). Primers are listed in S4 Table.

For HDV infection, PH cells were infected with 200 HDV genomes per cell (MOI 200) in the presence of 4% PEG8000 (Sigma-Aldrich, Missouri, USA). One day post-infection, cells were washed three times with PBS and treated further as indicated for the following experiment.

### AAV production and transduction

Adeno-associated virus (AAV) production was performed as described previously [56]. Briefly, HEK293T cells were seeded in a 150-mm plate, plasmids (pAAV-TTR-hNTCP, pAAV2/8-RC, and pHelper in 1:1:1 molar ratio) were co-transfected by polyethylenimine (PEI) (PolySciences, Pennsylvania, USA). 72 hours post-transfection, the supernatants and cells were harvested and filtered with 0.22 μm filter, then concentrated with 100-kDa Ultra Centrifugal Filters (Millipore, Massachusetts, USA) and subsequently purified by iodixanol gradient ultracentrifugation. The titer of recombinant AAV was determined by qPCR using NTCP-specific primers. Primer sequences are listed in S4 Table.

For AAV transduction experiments, PHs were transduced with 60000 viral genomes (vg) per cell diluted in PMM in the presence of 4% PEG8000. 16 hours post-transduction, cells were washed thrice with PBS and changed with fresh PMM. 72 hours post-transduction, cells were infected with HBV.

### Analysis of HBV replication markers

The analysis of HBV replication markers was performed as described previously [56]. Briefly, the levels of HBeAg and HBsAg in the cell culture supernatants were determined by enzyme-linked immunosorbent assay (ELISA) (Kehua Bio-Engineering Co., Shanghai, China) according to the manufacturer’s instructions. HBV DNA from cell cultural supernatants and intracellular was quantified by qPCR using an HBV viral DNA quantitative fluorescence diagnostic kit (Sansure Biotech, Changsha, China).

### Real-time quantitative PCR quantification of cellular HBV and HDV RNA

Total RNA from infected cells was purified using RNApure Tissue & Cell Kit (Cowin Biotech, Beijing, China) and transcribed into cDNA by ReverTra Ace qPCR RT Master Mix (Toyobo biotech co., Osaka, Japan). Intracellular HBV pgRNA, HBV total RNA, and HDV RNA were detected by RT-qPCR using LightCycler 480 (Roche Diagnostics, Basel, Switzerland). Primers are listed in S4 Table.

### Immunofluorescence staining

Immunofluorescence staining of HDAg and HBsAg was performed as described [8]. Briefly, cells in well-plate were washed with PBS and then immediately fixed in 4% paraformaldehyde (Invitrogen, California, USA) for 24 hours. Cells were washed three times with PBS and permeabilized with 0.1% Triton X-100 (Sigma-Aldrich, USA) for 10 min at room temperature. Cells were then blocked with 10% goat serum (Boster Biological Technology Co., Ltd., China) for 1 hour at 37°C. The following primary antibodies were used to stain the cells overnight at 4°C: rabbit anti-HBsAg (Novus Biologicals, Colorado, USA), rabbit anti-HDAg (self-made), and mouse anti-HDAg (Kerafast, Boston, USA). Subsequently, the cells were incubated with secondary antibody Alexa Fluor 488 goat anti-rabbit IgG (Invitrogen, California, USA) or Alexa Fluor 488 goat anti-mouse IgG (Invitrogen, California, USA) for 1 hour at 37°C. The nucleus was stained with Hoechst33258 (Invitrogen, California, USA) for 10 min at room temperature. The cells were then analyzed using a fluorescence microscope (OLYMPUS, Tokyo, Japan). More information about the antibodies is listed in S5 Table.

### Western blotting

Protein sample from cells was obtained by different treatments. The cells were lysed by using lysing buffer (50 mM Tris-HCl [pH 7.5], 1 mM ethylene diamine tetraacetic acid (EDTA), 1 mM EGTA, 150 mM NaCl, 1% Triton-X-100, 2 mM dithiothreitol (DTT), 100 μM phenylmethylsulfonyl fluoride (PMSF) and 1 μg/ml proteinase inhibitors), the protein concentration was determined by Bradford Assay Kit (Bio-Rad, California, USA). In general, 30 μg protein samples were separated by SDS-PAGE gel electrophoresis and subsequently transferred to the PVDF membrane (Millipore, Massachusetts, USA). Following blocked with 5% skim milk in tris-buffered saline with 0.1% Tween 20 (TBST) before antibody incubation. The following primary antibodies were used: anti-HBc (Gene Tech, Shanghai, China) and anti-β-actin (Abclonal, Wuhan, China). Anti-rabbit IgG (HRP-linked Antibody) (Cell Signaling Technology, Massachusetts, USA) was used as the secondary antibody. Finally, the signals were detected using Immobilon Western chemiluminescent HRP substrate (Millipore, Massachusetts, USA) by a Luminescent Image Analyzer (Syngene, England). The information for antibodies is shown in S5 Table.

### Northern blotting

The extraction and Northern blotting analyses of HBV mRNAs were performed as described previously [8]. Briefly, cells were suspended in TRIzol reagent and then extracted by Ultrapure RNA extraction kit (CW0581M, CWBIO, Taizhou, China) according to the manufacturer’s instructions. 20 µg RNA was subjected to denaturizing 1% (wt/vol) agarose gel electrophoresis with 2% formaldehyde reagents and transferred onto an Amersham Hybond-N+ membrane (GE Healthcare Lifesciences, Pittsburgh, USA) by capillary siphon method. Then, the membrane was fixed by UV cross-linking and followed by hybridization with HBV probes. The HBV probes were prepared by Random Primer DNA Labeling Kit Ver. 2 (Takara Bio, Kyoto, Japan). Finally, the Hybridization signal was captured by a Typhoon FLA 9500 imager (GE Healthcare Lifesciences, Pittsburgh, USA).

### Southern blotting

Purification of core-associated HBV DNA was performed as described previously [57]. Briefly, cultural cells were suspended in 800 μl lysis buffer (50 mM Tris-HCl [pH7.4], 50 mM NaCl, 1 mM EDTA, and 1%NP-40) and incubated on ice for 10 min. The lysate was then centrifuged at 14,500 g for 3 min at 4°C. Then 8 μl 1 M MgCl_2_ and 8 μl 10 mg/ml DNase I were added into the supernatant and incubated at 37°C for 30 min. 40 μl 0.5 M EDTA [pH8.0], 20 μl 20 mg/ml proteinase K, and 80 μl 10% sodium dodecyl sulfate (SDS) were added and incubated at 55°C for 2 hours. Core-associated HBV DNA was further extracted with phenol and chloroform, followed by precipitation using isopropanol and sodium acetate.

A Hirt protein-free DNA extraction procedure was used to extract HBV cccDNA from cells as described previously [56]. Briefly, for cultural cells, the cells were suspended in 1.5 ml TE buffer (10:10) (10 mM Tris-HCl [pH7.4] and 10 mM EDTA) supplemented with 100 μl 10% SDS for 30 min at room temperature. Then, 400 μl 5 M NaCl was added to precipitate proteins and protein-associated DNA for at least 16 hours at 4°C. Hirt protein-free DNA was further extracted with phenol and chloroform, followed by precipitation using isopropanol and sodium acetate.

All obtained core-associated HBV DNA or Hirt DNA samples were separated by agarose gel electrophoresis and transferred onto an Amersham Hybond-N+ membrane (GE Healthcare Lifesciences, Pittsburgh, USA) by capillary siphon method. Then, the membrane was fixed by UV cross-linking and followed by hybridization with HBV probes. The HBV probes were prepared by Random Primer DNA Labeling Kit Ver. 2 (Takara Bio, Kyoto, Japan). Finally, the Hybridization signal was captured by a Typhoon FLA 9500 imager (GE Healthcare Lifesciences, Pittsburgh, USA).

### Transmission electron microscopy (TEM)

Transmission electron microscopy detection of HBV particles was performed as described previously [11]. Briefly, the cells were trypsinized and washed 3 times with precooled PBS and were collected with centrifugation. The pellets were fixed with 2.5% glutaraldehyde in PBS at room temperature for 2 hours. The fixed pellets were rinsed with PBS, 3 times for 10 min each and were treated with 1% osmium tetroxide in PBS for 1 hour. After 3 times rinsing, the pellets were dehydrated through a graded series of ethanol concentrations (50%, 70%, 90%, and 100%) for 10 min each prior to embedment with resin. The samples were sliced into 50 nm sections using Leica UC7 (Leica Microsystems, Wetzlar, Germany) and analyzed with the transmission electron microscope HITATI 7800 (HITACHI, Tokyo, Japan).

### Statistical analysis

Statistical analysis was performed by GraphPad Prism 8.0 software (GraphPad Software, USA). The data were presented as the mean ± SD. The statistical analyses were carried out using Student’s unpaired two-tailed t-test. *P* values < 0.05 were considered significant. * *p* < 0.05, ** *p* < 0.01, *** *p* < 0.001, and **** *p* < 0.0001.

Additional methods are shown in S1 Text.

## Supporting information

S1 Text Supplementary methods. (DOCX)

S1 Fig Morphology of primary hepatocytes after seeding. Culture of multiple primary hepatocytes from different species. The morphology of multiple primary hepatocytes from different species was assessed one day post-seeding using a fluorescent inverted microscope. Scale bar = 100 µm.
(TIF)

S2 Fig PCR amplification of the COI gene from different species. The PCR amplification products were identified by 1% agarose gel electrophoresis.
(TIF)

S1 Table Primer sequences used for COI gene amplification. (DOCX)

S2 Table PCR amplification, sequencing, and deposited GeneBank accession number of COI gene homologues. (DOCX)

S3 Table The DNA and amino acid sequences of Siberian hamster. (DOCX)

S4 Table Primer sequences for qPCR assay. (DOCX)

S5 Table Antibodies used for western blot and immunostaining. (DOCX)

## Abbreviations

AAV: adeno-associated virus
cccDNA: covalently closed circular DNA
DMEM: Dulbecco’s Modified Eagle Medium
dslDNA: double stranded linear DNA
EGF: epidermal growth factor
ETV: entecavir
HBc: hepatitis B core protein
HDV: hepatitis D virus
HBeAg: hepatitis B e antigen
HBsAg: hepatitis B surface antigen
HBV: hepatitis B virus
ITS: insulin-transferrin-selenium
MOI: multiplicity of infection
mRNA: messenger RNA
MyrB: myrcludex B
hNTCP: human sodium taurocholate cotransporting polypeptide
PEI: polyetherimide
pgRNA: pregenomic RNA
Poly (I:C): polyinosinic-polycytidylic acid
qPCR: quantitative polymerase chain reaction
rcDNA: relaxed circular DNA
ssDNA: single stranded DNA
TEM: transmission electron microscopy
vg: viral genome
