# Supplementary material for: Screening of different species reveals cat hepatocytes support HBV infection
Source: PLoS Pathog. 2025 Aug 4;21(8):e1013390. doi: 10.1371/journal.ppat.1013390 (PMC12333979; doi:10.1371/journal.ppat.1013390)
Supplement: S1 Text — (DOCX) [file ppat.1013390.s001.docx]

**S1 Text**

**Genomic DNA extraction, amplification, and sequencing**

All primary hepatocytes from different species were extracted for genomic DNA using TIANamp Genomic DNA Kit (Tiangen, Beijing, China). 100 ng genomic DNA was used for the amplification of mitochondrially encoded cytochrome c oxidase subunit I (COI). The complete mitogenome of the corresponding species downloaded from GenBank was used as a reference sequence for designing species-specific COI gene amplification primers (S1 Table). The PCR reaction mixture contained 1×Prime STAR Mix (Takara Bio, Japan), 1 μM of each primer, and 100 ng genomic DNA. The PCR cycle was as follows: pre-denaturing for 5 min at 98℃, followed by 30 cycles of denaturing for 30 s at 98℃, annealing for 30 s at 56℃, extension for 30 s at 72℃, and a final extension at 72℃ for 5 min. The PCR products were detected using 1% agarose gel electrophoresis, all remaining PCR products were purified using Universal DNA Purification Kit (Tiangen, Beijing, China).

Purified fragments were sequenced and assembled by Quintara Biosciences (Wuhan, China). The sequencing results were annotated via Basic Local Alignment Search Tool (BLAST) alignment from the National Center for Biotechnology Information (NCBI) to search for homologous sequences based on the highest values ​​obtained in the percentage of identity, query cover, and bit score. All sequence data were submitted to GeneBank from NCBI, the accession numbers were listed in S2 Table.

**Cloning of Siberian hamster NTCP sequence**

The cDNA of Siberian hamster was used for the amplification of NTCP-specific regions. PCR primers (forward: 5'-ATGGAGGTACACAATTTTTC-3', reverse: 5'-CTAAATTATCACATGACCAG-3') were designed based on database sequences for the different hNTCP homologues, including Mesocricetus auratus (Genbank number XM_005072814.4) and RNA-seq of Phodopus sungorus (SRR17223274). The PCR reaction mixture contained 1×Prime STAR Mix (Takara Bio, Japan), 1 μM of each primer, and 100 ng cDNA. The PCR cycle was as follows: pre-denaturing for 5 min at 98℃, followed by 30 cycles of denaturing for 30 s at 98℃, annealing for 30 s at 58℃, extension for 30 s at 72℃, and a final extension at 72℃ for 5 min. The PCR products were purified using 1% agarose gel electrophoresis and Universal DNA Purification Kit (Tiangen, Beijing, China). The obtained hNTCP coding regions were ligated into the plasmid of pCE2 TA/Blunt-Zero using the 5 min TA Blunt-Zero Cloning Kit (Vazyme, Nanjing, China) according to the manufacturer’s instructions. Positive clone was sequenced and assembled by Quintara Biosciences (Wuhan, China). The sequencing results were listed in S3 Table and submitted to GeneBank from NCBI (Genbank number PV827218.1).
