# Supplementary figures and images for: Screening of different species reveals cat hepatocytes support HBV infection
Source: PLoS Pathog. 2025 Aug 4;21(8):e1013390. doi: 10.1371/journal.ppat.1013390 (PMC12333979; doi:10.1371/journal.ppat.1013390)

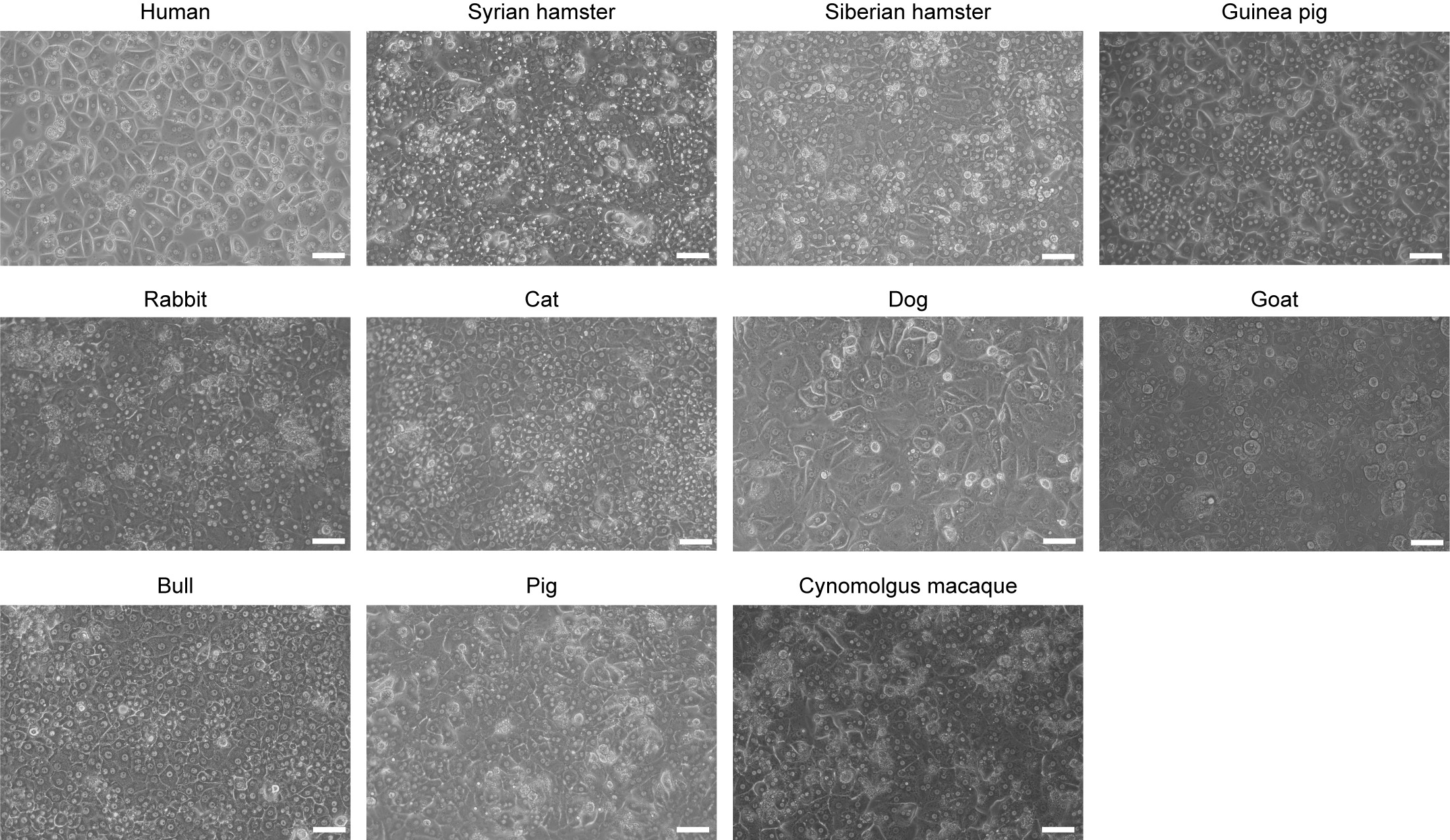

Supplement: S1 Fig — Culture of multiple primary hepatocytes from different species. The morphology of multiple primary hepatocytes from different species was assessed one day post-seeding using a fluorescent inverted microscope. Scale bar = 100 µm. (TIF) [file ppat.1013390.s002.tif]

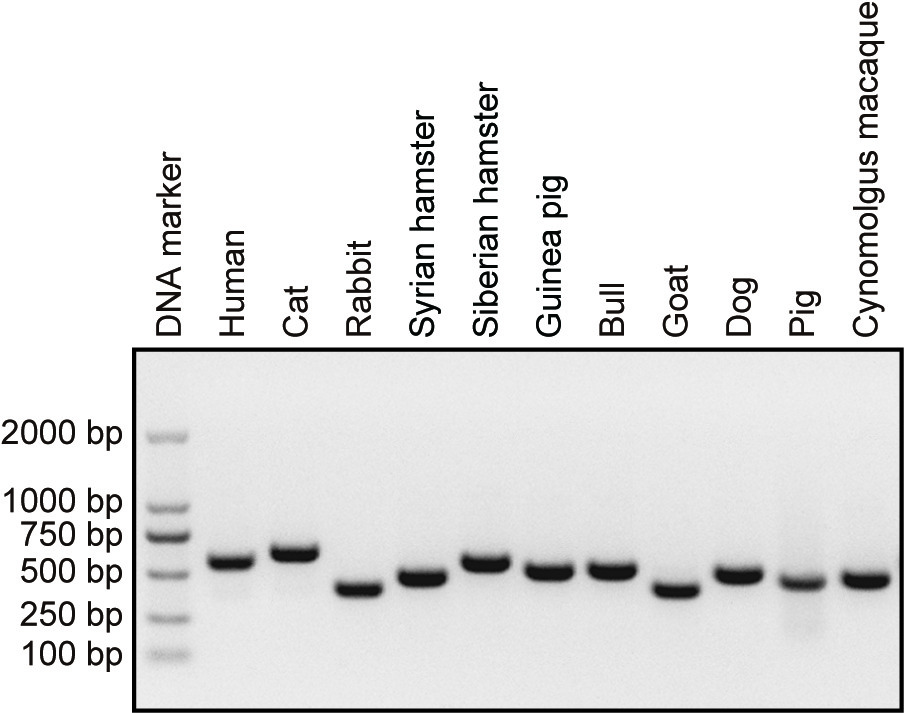

Supplement: S2 Fig — The PCR amplification products were identified by 1% agarose gel electrophoresis. (TIF) [file ppat.1013390.s003.tif]
