# Supplementary material for: Screening of different species reveals cat hepatocytes support HBV infection
Source: PLoS Pathog. 2025 Aug 4;21(8):e1013390. doi: 10.1371/journal.ppat.1013390 (PMC12333979; doi:10.1371/journal.ppat.1013390)
Supplement: S1 Table — (DOCX) [file ppat.1013390.s004.docx]

**S1 Table. Primer sequences used for COI gene amplification.**

| Species | Common name | Deriction | Sequence order (5'-3') |
| --- | --- | --- | --- |
| Homo sapiens | Human | Forward | CACACGAGCATATTTCACCTCC |
|  |  | Reverse | GGTTTATGGAGGGTTCTTCTACT |
| Felis catus | Cat | Forward | CTTAGCAGCGGGAATCACTATATTA |
|  |  | Reverse | TTGCTCAAGTGTTGTCAAGGG |
| Oryctolagus cuniculus | Rabbit | Forward | TTCGTCAATCGTTGACTTTTCTC |
|  |  | Reverse | CAGCATGTGCAAGATTACCG |
| Mesocricetus auratus | Syrian hamster | Forward | CCTACCGGGCTTCGGAATTA |
|  |  | Reverse | GCTTTTGCTCATGTATCGTCAAG |
| Phodopus sungorus | Siberian hamster | Forward | aaacttagcccacgcaggag |
|  |  | Reverse | ctccgtgaagggttgctagt |
| Cavia porcellus | Guinea pig | Forward | CGGAACACTCTTAGGCGATGAT |
|  |  | Reverse | CCCAGCAGGATCGAAAAAGG |
| Bos taurus | Bull | Forward | TTTGATGCTTGGGCCGGTAT |
|  |  | Reverse | CCGGCTGCTAATACAGGGAG |
| Capra hircus | Goat | Forward | ACTGCCGTACTACTCCTCCT |
|  |  | Reverse | CTCATATCATGGCGGGGGAC |
| Canis lupus familiaris | Dog | Forward | GCCCATCATAATTGGGGGCT |
|  |  | Reverse | CGAATCCGGGCAGGATAAGA |
| Sus scrofa | Pig | Forward | CACCCGCAATACTATGAGCTCTG |
|  |  | Reverse | TGTGTGATAGGGAGGAGGACA |
| Macaca fascicularis | Cynomolgus macaque | Forward | CACCCAGGAGCTTCTGTAGA |
|  |  | Reverse | ATTGCAATGATTATAGTGGC |
