# Supplementary material for: Screening of different species reveals cat hepatocytes support HBV infection
Source: PLoS Pathog. 2025 Aug 4;21(8):e1013390. doi: 10.1371/journal.ppat.1013390 (PMC12333979; doi:10.1371/journal.ppat.1013390)
Supplement: S2 Table — (DOCX) [file ppat.1013390.s005.docx]

**S2 Table. PCR amplification, sequencing, and deposited GeneBank accession number of COI gene homologues.**

| Species | Common name | PCR product length (bp) | Confident sequenced length (bp) | Genebank accession number |
| --- | --- | --- | --- | --- |
| Homo sapiens | Human | 575 | 538 | PV639009 |
| Felis catus | Cat | 629 | 609 | PV639086 |
| Oryctolagus cuniculus | Rabbit | 415 | 385 | PV639170 |
| Mesocricetus auratus | Syrian hamster | 495 | 468 | PV639248 |
| Phodopus sungorus | Siberian hamster | 587 | 549 | PV639326 |
| Cavia porcellus | Guinea pig | 541 | 507 | PV639402 |
| Bos taurus | Bull | 551 | 512 | PV639622 |
| Capra hircus | Goat | 451 | 423 | PV639632 |
| Canis lupus familiaris | Dog | 542 | 513 | PV639633 |
| Sus scrofa | Pig | 509 | 479 | PV639636 |
| Macaca fascicularis | Cynomolgus macaque | 530 | 473 | PV643198 |
