# Supplementary material for: Screening of different species reveals cat hepatocytes support HBV infection
Source: PLoS Pathog. 2025 Aug 4;21(8):e1013390. doi: 10.1371/journal.ppat.1013390 (PMC12333979; doi:10.1371/journal.ppat.1013390)
Supplement: S3 Table — (DOCX) [file ppat.1013390.s006.docx]

**S3 Table. The DNA and amino acid sequences of Siberian hamster.**

| Type of sequence | Sequence |
| --- | --- |
| DNA sequence | ATGGAGGTACACAATTTTTCAGCCCACTTAAATTTCTCTCTGCCGCCTGGCTTTGGCCACCGCCCCACAGACAGGGCGCTGAGTATTATCCTGGTAGTCATGCTGCTATTCGTCATGTTCTCACTCGGCTGCACCATGGAGTTCAGCAAGATCAAGGCTCACTTCTGGAAGCCCAAAGGGGTGGTCATTGCCATGTTGTCCCAGTATGGCATCATGCCCCTCACTGCCTTTGTCCTGGGCAAGGTCTTCCGTCTGAAACCAATTGAGGCACTGGCCATCCTCATCTGTGGCTGCTCTCCTGGGGGGAACCTGTCCAACCTCTTCACCCTGGCCATGAAGGGGGACATGAACCTCAGCATTGTGATGACCACCTGCTCCACCTTTGCTGCCTTGGGCATGATGCCTCTCCTCTTGTACATCTACACGAAAGGGATCTATGATGGCAATCTTAAGGACAAGGTGCCCTATGGAGGCATTATGATATCACTGGTCATGGTTCTCATTCCTTGTAGCATGGGGATCTTCCTCAAGACCAAAAAGCCACAGTATGTTCCCTACATCATCAAGGGAGGGGTAACCATCACTTTCTGCCTCAGTGTGGCTGTCACAGTTCTGTCTGTCATCAATGTGGGCAACAGCATCACATATGCCATGACACCACCATTACTGGCCACCTCCTCCCTGATGCCTTTCTCTGGCTTCCTGCTGGGCTACGTTCTCTCTGCTCTCTTCCAGCTCAGTCCAAGGTGCAGACGCACTATCAGCATGGAAACAGGATTCCAAAATGTCCAACTTTGTTCCACCATCCTCAATGTGACCTTCCCCCCTGAAGTCATTGGACCACTTTTCTTCTTTCCTCTTCTCTATATGATTTTTCAACTGTCAGAAGCAATTCTCTTCATTATTACCTTCCGGTGCTATGAGAAAATCAAGCCTTCTAAGGAGGAAAAAACCAAAGTGATCTACAAGGCTGCTGCAACTGAAGATGCTACTCCAGGGACTCTGGAAAAGGGCACCCACAATGGTAATATTTCTTCTACAAAACCTAGCCCTCCCCAATGGCCTGATTCTGGTCATGTGATAATTTAG |
| Amino acid sequence | MEVHNFSAHLNFSLPPGFGHRPTDRALSIILVVMLLFVMFSLGCTMEFSKIKAHFWKPKGVVIAMLSQYGIMPLTAFVLGKVFRLKPIEALAILICGCSPGGNLSNLFTLAMKGDMNLSIVMTTCSTFAALGMMPLLLYIYTKGIYDGNLKDKVPYGGIMISLVMVLIPCSMGIFLKTKKPQYVPYIIKGGVTITFCLSVAVTVLSVINVGNSITYAMTPPLLATSSLMPFSGFLLGYVLSALFQLSPRCRRTISMETGFQNVQLCSTILNVTFPPEVIGPLFFFPLLYMIFQLSEAILFIITFRCYEKIKPSKEEKTKVIYKAAATEDATPGTLEKGTHNGNISSTKPSPPQWPDSGHVII* |
