# Supplementary material for: Screening of different species reveals cat hepatocytes support HBV infection
Source: PLoS Pathog. 2025 Aug 4;21(8):e1013390. doi: 10.1371/journal.ppat.1013390 (PMC12333979; doi:10.1371/journal.ppat.1013390)
Supplement: S4 Table — (DOCX) [file ppat.1013390.s007.docx]

**S4 Table. Primer sequences for qPCR assay.**

| Oligo name | Sequence order (5′–3′) |
| --- | --- |
| HBV pgRNA-fwd | CTGGGTGGGTGTTAATTTGG |
| HBV pgRNA-rev | TAAGCTGGAGGAGTGCGAAT |
| HBV total RNA-fwd | CCGTCTGTGCCTTCTCATCTGC |
| HBV total RNA-rev | ACCAATTTATGCCTACAGCCTCC |
| HDV RNA-fwd | CGGAGATGAGCAGAGAAATCA |
| HDV RNA-rev | ACTCCTAGCATCTCCTCCTATC |
| hNTCP 95-fwd | TGTTCATGTTGTTCTTCATC |
| hNTCP 270-rev | TGCCTCAATGTTCTTCAGCC |
| Cat ACTB 821-fwd | TCCACGAAACTACCTTCAACTC |
| Cat ACTB 955-rev | CAGTGATCTCCTTCTGCATCC |
