# Supplementary material for: Screening of different species reveals cat hepatocytes support HBV infection
Source: PLoS Pathog. 2025 Aug 4;21(8):e1013390. doi: 10.1371/journal.ppat.1013390 (PMC12333979; doi:10.1371/journal.ppat.1013390)
Supplement: S5 Table — (DOCX) [file ppat.1013390.s008.docx]

**S5 Table.** **Antibodies used for western blot and immunostaining.**

| Antibody | Source | Cat# | Host | Dilution |
| --- | --- | --- | --- | --- |
| β-actin | Abclonal | AC038 | Rabbit | 1:10000  (Western blotting) |
| Anti-HBsAg | Novus Biologicals | NB100-62652 | Rabbit | 1:200  (Immunofluorescence staining) |
| Anti-HBc | self-made | N/A | Rabbit | 1:1000  (Western blotting) |
| Anti-HDAg | self-made | N/A | Rabbit | 1:200  (Immunofluorescence staining) |
| Anti-HDAg | Kerafast | EHD001 | Mouse | 1:100  (Immunofluorescence staining) |
| Anti-hNTCP serum K9 | Gifted from Prof. Bruno Stieger | N/A | Rabbit | 1:1000  (Western blotting) |
| Anti-rabbit IgG (HRP-linked Antibody) | Cell Signaling Technology | 7074S | Goat | 1:10000  (Western blotting) |
| Anti-rabbit IgG (Alexa Fluor 488) | Invitrogen | A-11034 | Goat | 1:1000  (Immunofluorescence staining) |
| Anti-mouse IgG (Alexa Fluor 488) | Invitrogen | A-11029 | Goat | 1:1000  (Immunofluorescence staining) |
